# Lipid Metabolism and Circadian Regulation in Wing Polyphenism of *Rhopalosiphum padi*: Transcriptomic Validation of Key DEGs for Biocontrol

**DOI:** 10.3390/genes16101163

**Published:** 2025-09-30

**Authors:** Yan Zhang, Tao Zhang, Jianwu Mao, Shenhang Cheng

**Affiliations:** 1 Department of Biological Science and Technology, Jinzhong University, Jinzhong 030619, China; jzxyvm1000224@jzxy.edu.cn; 2 School of Life Science, Shanxi University, Taiyuan 030006, China; 3 School of Synthetic Biology, Shanxi University, Taiyuan 030006, China; zhangtao12@sxu.edu.cn (T.Z.); maojianwu@sxu.edu.cn (J.M.)

**Keywords:** *Rhopalosiphum padi*, wing polyphenism, transcriptomics, lipid metabolism, circadian rhythm, energy allocation, RNAi-based biocontrol

## Abstract

Background/Objectives: The bird cherry-oat aphid, *Rhopalosiphum padi*, is a major global pest of cereal crops and exhibits wing polyphenism, producing both winged (dispersive) and wingless (reproductive) morphs. Methods: To identify potential RNAi targets that could specifically disrupt the migratory winged morph, we conducted a comparative transcriptomic analysis of adult aphids. Differentially expressed genes (DEGs) were identified, annotated for their functions, and analyzed for their involvement in metabolic pathways. Results: Significant differences were observed in 121 genes between morphs: 13 were upregulated in the winged morph, while 108 were downregulated. Most DEGs were enriched in lipid metabolism and circadian rhythm pathways, suggesting that wing polymorphism may be adaptively linked to energy resource allocation strategies. Conclusions: This study firstly reveals the adult-stage-specific regulatory roles of lipid metabolism and circadian rhythm pathways in wing polyphenism, identifying six candidate genes (*BCORL1*, *AMP-L*, *Pfl*, *Lip3L*, *HLFL(X7)*, and *HLFL(X4)*) for RNAi-based biocontrol strategies targeting migratory morphs.

## 1. Introduction

Insects are the earliest animal group to have evolved wings, and the development of flight capability profoundly shaped their behavioral adaptations to the environment. Many insect species exhibit non-genetic wing polyphenism, wherein a single genotype displays two or more distinct morphotypes. Aphids represent a paradigmatic example of wing polyphenism, featuring fully winged and completely wingless forms within clonal populations—a plasticity regulated by transgenerational epigenetic mechanisms rather than genetic variation [1]. This polyphenism represents an evolutionary trade-off between dispersal and reproduction: wingless morphs prioritize energy investment in offspring production, while winged individuals sacrifice fecundity for the long-distance dispersal capabilities necessary to colonize optimal habitats [2,3].

Evolutionarily, wing plasticity enables aphids to evade environmental stressors while facilitating the spread of plant viruses, posing significant agricultural risks [4]. Research on aphid wing polyphenism has focused on two axes: (1) Environmental cues—population density, temperature, photoperiod, and host plant quality [2,5,6,7,8,9]; (2) Endocrine regulation—ecdysteroid, juvenile hormone, and insulin/IGF signaling pathways [10,11,12,13,14,15,16,17]. Recent studies have identified the roles of key developmental signaling pathways, such as Wnt, in wing morph determination during early nymphal stages [18], as well as post-transcriptional regulation by microRNAs like *miR-147b* targeting vestigial (*vg*) to modulate wing differentiation [19]. Moreover, miRNA-mediated regulatory networks, including *miR-9b-ABCG4*-insulin signaling, have been shown to transduce crowding signals into wing dimorphism in *Aphis citricidus* and *Acyrthosiphon pisum* [7], while temporal miRNA profiling in *Sitobion avenae* revealed stage-specific roles for *miR-2*, *miR-14*, and *miR-306* in wing development [20]. Additionally, long non-coding RNAs (lncRNAs) have emerged as critical regulators of wing development, as demonstrated in *Aphis citricidus*, where *Ac_lnc54106.1* targets *PGBD4* and influences wing patterning genes [21].

The bird cherry-oat aphid (*Rhopalosiphum padi*; Hemiptera: Aphididae) is a major pest of cereal crops worldwide, and exemplifies this adaptive strategy. Wingless morphs exhibit enlarged ovaries for rapid parthenogenesis, whereas winged forms develop flight muscles for migration [22], but adult-specific metabolic adaptations are unexplored. Moreover, recent studies in other aphid species such as *Acyrthosiphon pisum* have revealed that alternative splicing plays a crucial role in generating proteomic diversity between polyphenic morphs, independent of differential gene expression [23]. This suggests that post-transcriptional regulatory mechanisms may also contribute significantly to wing polyphenism, an aspect yet to be investigated in *R. padi*.

Here, we integrate RNA-seq and RT-qPCR to identify DEGs between winged/wingless adult *R. padi* from wheat fields in Shanxi, China. Our study advances three key insights: (1) Lipid metabolism pathways dominate adult wing maintenance, contrasting with nymphal signaling pathways such as Wnt and insulin/ecdysone; (2) Circadian rhythm genes link environmental cues to morph persistence; (3) Six validated DEGs (e.g., *Lip3L*, *HLFLs*) offer RNAi targets for biocontrol. Additionally, we contextualize our findings within the broader framework of miRNA-regulated wing development, suggesting potential cross-talk between metabolic and post-transcriptional regulatory layers. Furthermore, we draw parallels with recent findings in *Aphis gossypii*, where insulin signaling and lipid metabolism are also central to wing dimorphism across multiple winged morphs [24], highlighting conserved yet species-specific regulatory strategies. This work bridges the gap between developmental signaling and adult metabolic phenotypes, providing a framework for stage-specific pest management. The findings will not only advance our understanding of the molecular mechanisms underlying wing polyphenism, but also identify key genes for potential RNAi-based biocontrol strategies against this pest.

## 2. Materials and Methods

### 2.1. Materials

The initial population of *R. padi* (wheat aphid) was collected from Yuncheng, Shanxi Province. Both winged and wingless adult morphs were used for experiments. All samples were collected within 2 h after light onset to standardize circadian effects on gene expression.

Wheat cultivar Jinchun 6 was purchased from Hebei Qingfeng Seed Co., Ltd., (Hengshui City, China).

All chemical reagents were of analytical grade unless otherwise specified. Total RNA was extracted using RNA extraction kits (Promega, Shanghai, China) or Trizol reagent (Takara, Beijing, China), followed by cDNA synthesis with PrimeScript™ II 1st Strand cDNA Synthesis Kit (Takara, Beijing, China). Nucleic acid purification was performed using the Gel Extraction Kit (Omega Bio-tek, Norcross, GA, USA) and AMPure XP beads (Omega Bio-tek, Norcross, GA, USA). PCR amplification utilized 2 × Rapid Taq Master Mix (Vazyme Biotech, Nanjing, China), while quantitative PCR was conducted with BlasTaq™ 2 × qPCR Master Mix (abm, Richmond, BC, Canada). Sample incubation was carried out in an artificial climate chamber (Jiangnan Instrument Factory, Ningbo, China), with morphological observations under an ES-18TZLED stereo microscope (Motic, Xiamen, China). Nucleic acid concentration was measured by NanoDrop 2000 spectrophotometer (Thermo Scientific, Wilmington, NC, USA), and real-time PCR analysis was performed on a CFX96 system (Bio-Rad, Hercules, CA, USA). High-throughput sequencing was executed on the NovaSeq™ 6000 platform (Illumina, San Diego, CA, USA).

### 2.2. Methods

#### 2.2.1. Total RNA Extraction from *R. padi*

Under a stereo microscope, 200 individuals of 3-day-old winged and wingless *R. padi* adults were collected per sample (three replicates per morph). Total RNA was extracted using the Trizol method following the manufacturer’s protocol (RNA extraction kit). RNA quality was assessed, and qualified samples were stored at −80 °C for further use.

#### 2.2.2. cDNA Library Construction and Transcriptome Sequencing

Transcriptome sequencing was performed by Sangon Biotech (Shanghai, China). After quality control, poly(A)-tailed mRNA was enriched using oligo(dT) beads. Purified mRNA was fragmented using divalent cations, and double-stranded cDNA was synthesized using random hexamer primers. The cDNA was end-repaired, A-tailed, and ligated with sequencing adapters. Size-selected fragments were PCR-amplified to construct the final sequencing library.

Libraries were pooled based on effective concentration and sequenced on the Illumina NovaSeq 6000 platform (PE150 mode). The sequencing principle relies on Sequencing by Synthesis (SBS): Fluorescently labeled dNTPs, DNA polymerase, and adapter primers are incorporated into the flow cell. As each cluster extends, fluorescence signals are captured and converted into base calls.

#### 2.2.3. Transcriptome Assembly and Functional Annotation

Raw reads were processed with Trimmomatic to remove adapters, poly-N sequences, and low-quality reads (Q ≤ 20 for >50% of bases). High-quality clean reads were assessed for Q20, Q30, and GC content (Q20 > 95%, Q30 > 95%, GC content ~34%).

De novo assembly was performed using Trinity, generating a total of 108,632 unigenes with an average length of 666.54 bp. Functional annotation was conducted by aligning transcripts against multiple databases, including NR (non-redundant protein), NT (nucleotide sequence), SwissProt (curated protein sequences), KOG (eukaryotic ortholog groups), GO (Gene Ontology), PFAM (protein families), CDD (conserved domains), and KEGG (Kyoto Encyclopedia of Genes and Genomes). These databases provide comprehensive functional and evolutionary insights. Detailed annotation rates are summarized in Table S2.

#### 2.2.4. Differential Gene Expression (DEG) Analysis

DESeq was used to identify DEGs between winged and wingless morphs (threshold: q-Value < 0.05, |Fold Change| > 2). Functional enrichment analysis was performed using GO and KEGG databases to identify biologically relevant pathways. Enrichment was calculated as the ratio of DEGs annotated to a pathway versus all annotated genes.

#### 2.2.5. Validation of DEGs by RT-qPCR

To confirm the reliability of transcriptome sequencing data, six differentially expressed genes (DEGs) exhibiting significant expression variations (|log_2_FC| > 2, *p* < 0.01) were randomly selected for experimental validation. The housekeeping gene β-actin (GenBank accession: MF083568) was used as an internal control. Six DEGs were selected based on KEGG pathway dominance: two lipid metabolism (*Lip3L*, *PLB1L*), two circadian rhythm (*HLFLs*), one reproduction (*Pfl*), and one stress response (*BCORL1*). Primer sequences are listed in Table 1.

cDNA synthesis. Total RNA (5 μg) extracted from each biological replicate was reverse-transcribed using the PrimeScript™ II 1st Strand cDNA Synthesis Kit (Takara Bio, Cat#6210A) following the manufacturer’s protocol. RNA integrity was verified by agarose gel electrophoresis (RIN ≥ 7.0) and quantified via NanoDrop 2000 spectrophotometer (Thermo Fisher Scientific).

qPCR assay. Reaction system: Each 20 μL reaction contained 10 μL BlasTaq™ 2 × qPCR Master Mix (Applied Biosystems, Cat#A25742, Waltham, MA, USA), 4 μL diluted cDNA (1:10), 0.8 μL each of gene-specific primers (10 μM), and 4.44 μL nuclease-free water.

Primer design. All primers were designed using Premier 5.0 software (Premier Biosoft) with the following criteria: amplicon length 80–150 bp, Tm = 60 ± 2 °C, GC content 40–60%. Primer specificity was confirmed by melt curve analysis and electrophoresis (Sangon Biotech, Shanghai).

Amplification protocol. Reactions were performed on a QuantStudio 6 Flex system (Applied Biosystems) under the following conditions: initial denaturation at 95 °C for 30 s; 40 cycles of 95 °C for 5 s and 60 °C for 34 s (fluorescence acquisition); followed by melt curve analysis (95 °C→60 °C→95 °C, 0.5 °C/s increments).

Gene expression levels were quantified using the 2^−ΔΔCT^ method. Triplicate measurements were applied for the gene. Each sample was analyzed in two technical replicates.

### 2.3. Data Analysis

Statistical analyses were performed using Microsoft Excel 2019 for data organization and GraphPad Prism 9.0 (GraphPad Software, San Diego, CA, USA) for statistical computations. Significant differences between experimental groups were determined by one-way ANOVA followed by Sidak’s multiple comparisons, with *p* < 0.05 considered statistically significant. Data are presented as mean ± SEM, unless otherwise specified.

## 3. Results and Analysis

### 3.1. Transcriptome Sequencing Data of Winged and Wingless R. padi

All sequencing data met high-quality standards, with Q30 scores exceeding 96.68%. After assembly, a total of 108,632 unigenes were generated, with an average length of 666.54 bp. Functional annotation revealed that the NR and NT databases provided the broadest coverage (40.59% and 46.91%, respectively), while KEGG and CDD annotations were less comprehensive, suggesting potential aphid-specific gene functions. Full statistics are available in Tables S1 and S2 and Figure S1.

### 3.2. Differentially Expressed Genes (DEGs) Between Winged and Wingless R. padi

**Figure 1:**
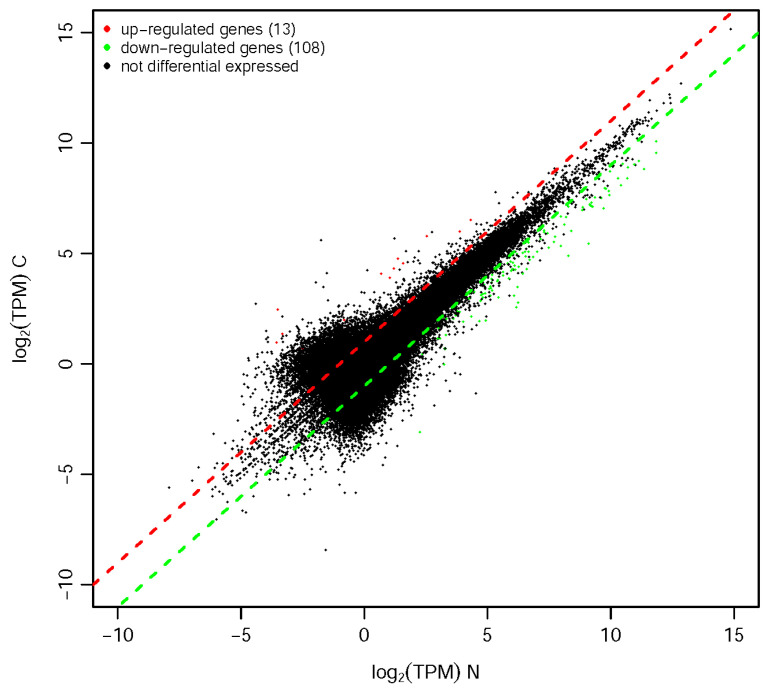
Scatter plot of expression differences between comparison groups. The x- and y-axes represent the log_2_(TPM) values of the two sample groups. Each point corresponds to a gene, with points closer to the origin indicating lower expression levels. Red points denote upregulated genes, green points represent downregulated genes, and black points indicate non-differentially expressed genes. Red/green dots denote DEGs (FDR < 0.05, |log_2_FC| > 1); dashed lines indicate significance thresholds. Upregulation/downregulation is defined as the log_2_(TPM) ratio of the y-axis group relative to the x-axis group. “C” indicates winged morph, while “N” denotes wingless morph.

Comparative transcriptome analysis revealed 121 significantly differentially expressedd genes (DEGs) between winged morphs and wingless morphs ((|log_2_FC| ≥ 1, FDR < 0.05), including 13 upregulated and 108 downregulated in the winged morph compared to the wingless morph (Figure 1). Notably, the downregulated genes predominated, suggesting potential suppression of specific pathways in winged morphs.

### 3.3. Functional Enrichment of DEGs in Wing Morph Differentiation

**Figure 2:**
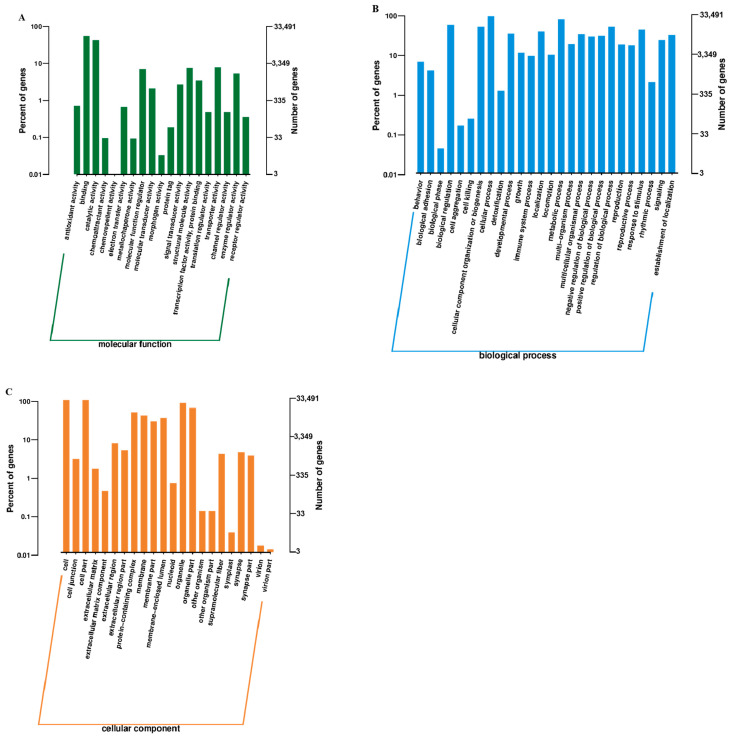
Gene Ontology (GO) classification of differentially expressed genes (DEGs) between winged and wingless morphs of *R. padi*. (**A**) Biological Process, (**B**) Cellular Component, and (**C**) Molecular Function. Light-colored bars represent the proportion of DEGs relative to all genes annotated in that category (left y-axis), while dark-colored bars indicate the total number of genes per category (right y-axis).

**Figure 3:**
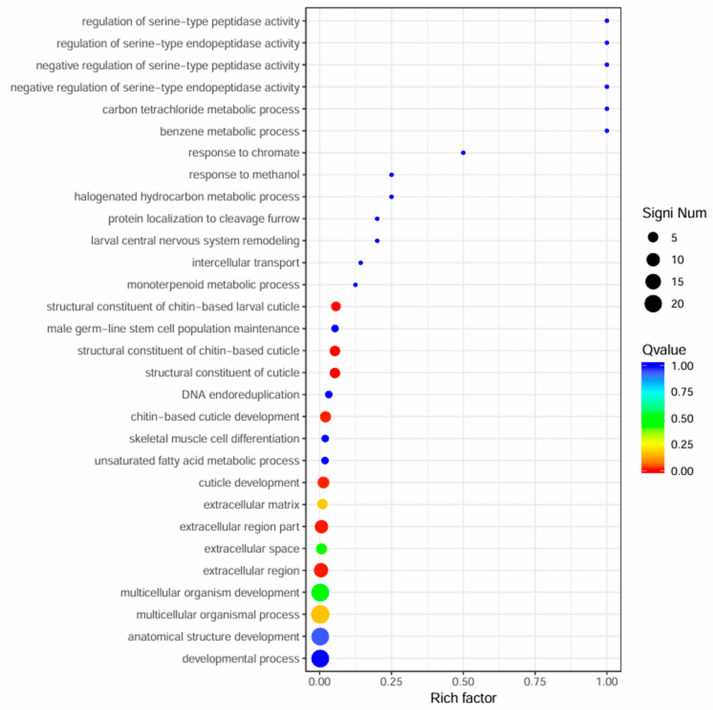
Scatter plot of significant GO enrichment analysis for differentially expressed genes between winged and wingless morphs of *R. padi.* The y-axis represents functional annotation categories, while the x-axis indicates the Rich Factor for each GO term. The Rich Factor is calculated as the ratio of differentially expressed genes annotated to a specific term to the total number of genes annotated in that GO category. A higher Rich Factor indicates a greater degree of enrichment. The color gradient of each point reflects the q-value (smaller q-values appear more red), and the size of each point corresponds to the number of differentially expressed genes in a given term (larger points indicate more genes).

GO enrichment analysis showed that DEGs were primarily associated with cuticle development and extracellular structure organization (Figure 2A–C and Figure 3, Table 2). Key terms included structural constituents of the cuticle and chitin-based larval cuticle, indicating roles in morphological adaptation.

### 3.4. KEGG Pathway Enrichment Analysis of DEGs

**Figure 4:**
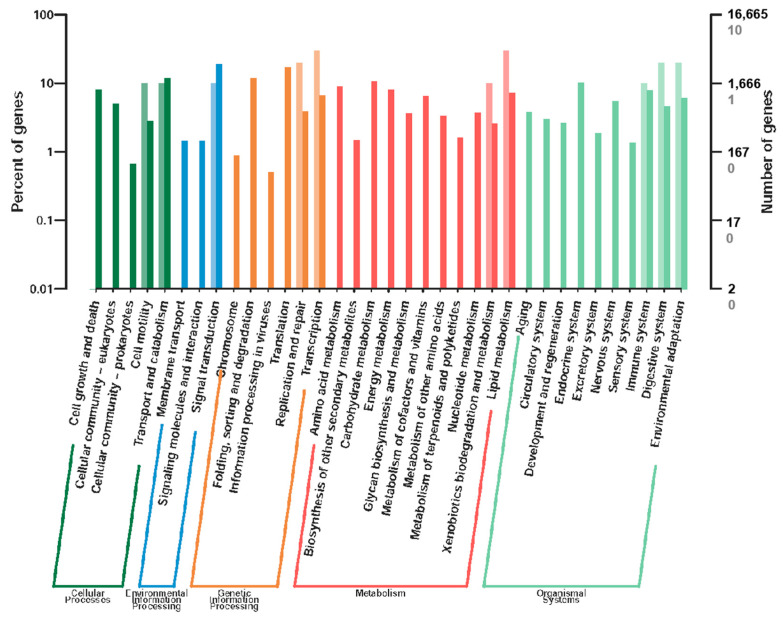
KEGG functional classification of differentially expressed genes (DEGs) between winged and wingless morphs of *R. padi.* The x-axis represents functional categories, with different colors indicating distinct classifications. Light-colored bars and values on the y-axis correspond to DEGs, while dark-colored bars and values represent all annotated genes. The right y-axis indicates the number of genes per category, whereas the left y-axis displays the proportion of DEGs relative to all genes annotated within each functional category.

**Figure 5:**
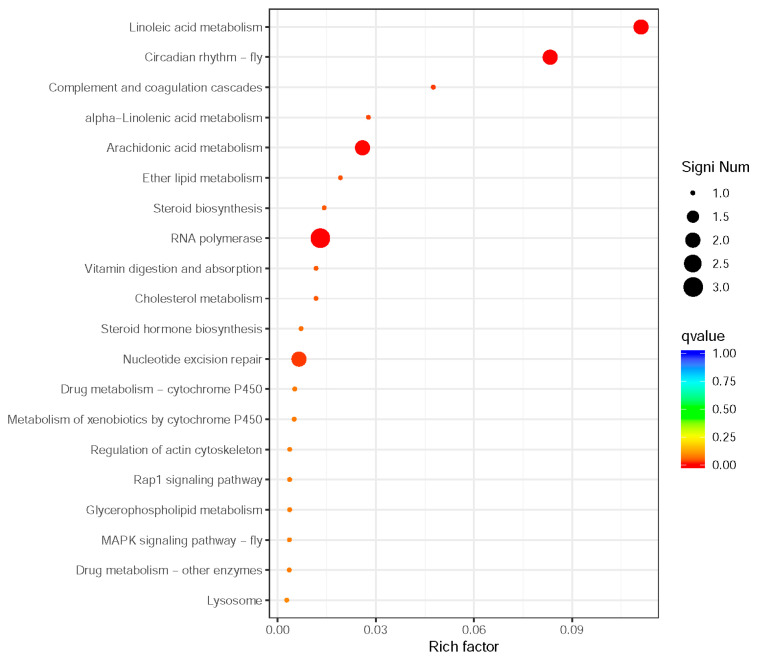
Scatter plot of KEGG functional enrichment analysis for differentially expressed genes between winged and wingless morphs of *R. padi.* The y-axis lists pathways, while the x-axis plots enrichment significance (Rich Factor: DEGs in a pathway/total annotated genes in that pathway). Points are colored by q-value (red = low q-value, high significance) and sized by DEG count.

KEGG analysis (q-value < 0.05) revealed significant enrichment in lipid metabolism pathways (e.g., linoleic acid and arachidonic acid metabolism) and circadian rhythm pathways (Figure 4 and Figure 5). These pathways are critical for energy allocation and environmental adaptation. Other enriched pathways included nucleotide excision repair and immune-related processes, reflecting trade-offs between dispersal and reproduction.

Notably, lipid metabolism pathways, including linoleic acid (2 genes), arachidonic acid (2 genes), α-linolenic acid (1 gene), and ether lipid metabolism (1 gene), were prominently enriched, suggesting their potential roles in maintaining cuticle flexibility and providing energy reserves for sustained flight. Two circadian rhythm-related genes were identified, indicating possible temporal regulation of wing development and dispersal behaviors. Enhanced DNA maintenance capacity was suggested by the enrichment of nucleotide excision repair (2 genes) and RNA polymerase (3 genes) pathways, which may contribute to genomic stability during morph differentiation. Immune-related pathways such as complement and coagulation cascades (1 gene), along with steroid biosynthesis (1 gene) and cholesterol metabolism (1 gene), potentially reflect physiological trade-offs between immune defense and reproductive investment in wing-dimorphic aphids. Additionally, the vitamin digestion and absorption pathway (1 gene) highlights nutritional adaptation strategies that may differ between morphs. Six significantly differentially expressed genes were screened and annotated in the Aphididae database, revealing their potential functional roles in insect resistance or stress response (Table 3).

To ensure analytical rigor, we implemented strict statistical thresholds (q-value < 0.05) and provided detailed gene counts for each enriched pathway. The functional clustering of pathways (e.g., lipid metabolism grouped with cuticle development) enhances the mechanistic interpretation of wing polyphenism. These findings collectively demonstrate comprehensive metabolic adaptations underlying wing polyphenism, with particular emphasis on energy allocation, stress response, and developmental regulation.

### 3.5. Functional Validation of Differentially Expressed Genes

**Figure 6:**
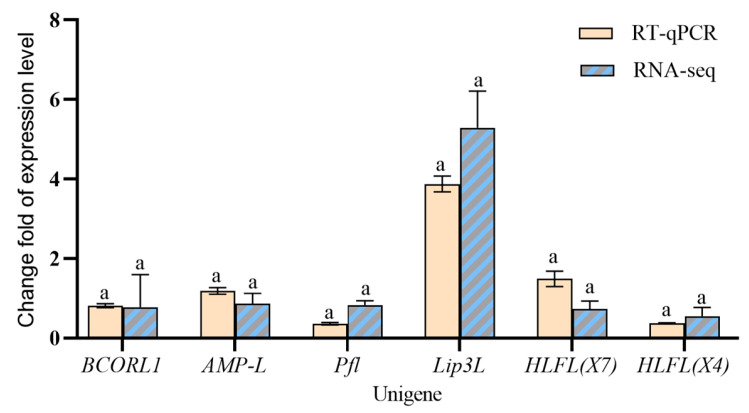
Differentially expressed genes between winged and wingless *R. padi* verified by RT-qPCR. Data are mean ± SEM. Same lowercase letters for the same gene indicate a non-significant difference at the *p* < 0.05 level, analyzed by two-way ANOVA test.

The expression levels of six significantly differentially expressed genes were validated by RT-qPCR (*F* = 1.468, *p* = 0.2375 > 0.5, 95% CI: −0.4208 to 0.1095). Statistical analysis showed no significant difference between technical replicates (Figure 6), supporting the reliability of the transcriptomic results.

## 4. Discussion

Transcriptome sequencing of winged and wingless adult *R. padi* using Trinity assembly yielded 12,570 unigenes, notably fewer than the 39,699 unigenes reported in 3rd-instar nymphs [22] and the 39,328 unigenes identified across all developmental stages by Zhang Q. et al. [25]. The adult-specific downregulation suggests a metabolic shift from developmental plasticity to flight/reproduction trade-offs, contrasting with nymph-stage energy allocation patterns [22]. This aligns with Zhang R. et al. (2019)’s observation that wing-patterning genes (e.g., *Wnt2*, *Dpp*, *Foxo*) peak in expression during early nymphal stages but decline in adults [3], indicating that developmental signaling cascades initiate morph determination, while adult phenotypes are maintained through metabolic reprogramming. This is consistent with findings in *A. pisum*, where Wnt signaling and apoptosis regulate wing primordia fate in early instars [18], while adult wing maintenance relies on metabolic pathways such as lipid metabolism, as highlighted in our study. Furthermore, recent studies indicate that miRNAs such as miR-9b and miR-147b fine-tune wing development through targeting key genes like *ABCG4* and *vg* [7,19], suggesting that post-transcriptional regulation may complement metabolic adaptations in maintaining wing phenotypes. Similarly, lncRNAs have been shown to regulate wing development in *Aphis citricidus* via targeting transposable element-derived genes such as *PGBD4* [21], underscoring the multi-layered regulatory complexity of wing plasticity. This aligns with ecological adaptation but requires validation via temporal expression profiling. This reduced transcriptomic complexity in adults may reflect developmental shifts in energy allocation priorities, feeding behavior adjustments, and metabolic reprogramming to accommodate distinct ecological functions [26,27].

Differential expression analysis identified only 121 significantly dysregulated genes in winged adults compared to wingless morphs, with a clear predominance of downregulation. This pattern aligns with findings in *R. padi* nymphs [22], but contrasts with the dynamic expression patterns of biogenic amine/hormone receptors (e.g., SeT, EcR, JHE) observed by Zhang R. et al. across instars [3]. This suggests that while early stages prioritize signal perception and transduction, adults focus on executing energy allocation strategies. In addition, this contrasts with studies on *Lipaphis erysimi* (upregulated genes dominant) and *Myzus persicae* (inconsistent trends) [25], suggesting species-specific regulatory conservation in wing polyphenism. Notably, the limited number of DEGs in adults may also reflect the involvement of post-transcriptional regulators such as miRNAs, which could modulate gene expression without transcriptional changes, as demonstrated in *S. avenae* [20].

Our KEGG pathway analysis identified 10 significantly enriched metabolic pathways (q-value < 0.05), demonstrating that lipid metabolism (linoleic/arachidonic acid) and circadian regulation dominate adult wing morph maintenance—a shift from the *Wnt*/*Notch*/*Dpp* signaling pathways prevalent in nymphs [3]. These findings are consistent with the metabolic reprogramming hypothesis, wherein adult aphids prioritize energy storage and utilization over developmental signaling. These pathways, including linoleic acid metabolism (2 genes), circadian rhythm-fly (2 genes), arachidonic acid metabolism (2 genes), RNA polymerase (3 genes), and nucleotide excision repair (2 genes), provide compelling evidence that wing polyphenism represents an adaptive energy allocation strategy in response to environmental stressors. Lipids, serving as primary energy storage molecules and essential energy sources, play a crucial role in insect flight, reproduction, and adaptation to environmental changes [28,29,30]. Therefore, the association between lipid metabolism and wing morph differentiation holds significant biological importance. Linoleic acid metabolites can activate or inhibit the activity of certain transcription factors, thereby influencing wing growth and differentiation [31,32]. This is further supported by studies in *A. gossypii*, where lipid and carbohydrate metabolism pathways are significantly upregulated across all three winged morphs compared to wingless females, highlighting a conserved metabolic basis for wing maintenance [24].

The enrichment of lipid metabolism pathways (linoleic, α-linolenic, and ether lipid metabolism) suggests these pathways serve as critical energy reservoirs for flight capacity, while circadian rhythm regulation likely mediates density- and temperature-dependent developmental switches. Linoleic acid metabolism (*Lip3L*) may regulate cuticle flexibility to support flight, complementing Zhang R. et al.’ s finding that cuticle development genes (e.g., *Ap*, *Dll*) are upregulated in early wing morphs [3]. Similarly, the circadian function of *HLFL*(X7) could mediate wing induction in response to population density [7], whereas nymphal stages rely on insulin/ecdysone crosstalk for morph commitment [3]. Notably, the downregulation of *BCORL1* in winged morphs may reflect its role as a transcriptional corepressor in Polycomb complexes, potentially repressing wing development genes in wingless morphs—a mechanism warranting further epigenetic investigation. Notably, *R. padi* exhibits stronger enrichment in lipid metabolism pathways compared to *M. persicae* [33], implying species-specific energy allocation strategies for winged morph development.

Complementary pathways such as complement/coagulation cascades (1 gene), steroid biosynthesis (1 gene), and cholesterol metabolism (1 gene) indicate physiological trade-offs between immune function and reproductive investment. The vitamin digestion/absorption pathway (1 gene) further highlights nutritional adaptations supporting divergent life history strategies. Collectively, these findings demonstrate that wing polyphenism fundamentally involves metabolic plasticity, with lipid metabolism providing the energetic foundation and circadian systems integrating environmental cues to optimize the dispersal-reproduction trade-off. This aligns with previous findings in 3rd instar nymphs [22], reinforcing that material and energy supply are key drivers of morph differentiation in aphids.

The six validated DEGs (*BCORL1*, *AMP-L*, *Pfl*, *Lip3L*, *HLFL*(X7), and *HLFL*(X4)) represent novel targets for RNAi-based biocontrol aimed at disrupting wing morph stability in adult *R. padi*. For example, silencing *Lip3L* could compromise lipid reserves essential for flight, while targeting *HLFL(X7/X4)* may disrupt circadian-regulated developmental timing, effectively inhibiting the formation of migratory morphs. This strategy capitalizes on the metabolic and regulatory specificity of the adult stage, offering a means to suppress dispersal and virus transmission without directly impacting wingless aphids’ reproduction, offering a sustainable management advantage.

Notably, the downregulation of *BCORL1* in winged adults suggests a potential role as a repressor of wing development in wingless morphs, possibly through Polycomb-mediated epigenetic mechanisms [34]. Similarly, *HLFL(X7/X4)* may interface with ecdysteroid signaling, indicating a conserved hormonal axis across life stages [3,12]. The potential regulation of these genes by miRNAs, as observed in other aphid species where miR-9b targets *ABCG4* [7], and the involvement of lncRNAs in fine-tuning wing development genes, as shown in *A. citricidus* [21], warrant further investigation to elucidate the multi-level regulatory network governing wing polyphenism.

Translating these findings into a viable field strategy, however, necessitates addressing key challenges in stability, delivery, and commercial scalability [35]. A primary limitation is the environmental instability of naked dsRNA. To overcome this, formulation advances—such as encapsulation in clay nanosheets or peptide-based carriers—have been shown to prolong dsRNA persistence on leaf surfaces and enhance cellular uptake in other insect pests [36,37]. For delivery, foliar sprays offer immediate application potential, while plant-mediated RNAi (e.g., expressing target dsRNAs in wheat) could provide continuous protection but requires significant investment in transgenic development and regulatory approval [38]. From a commercial perspective, the scalability of dsRNA production is critical. While recent advances in fermentation-based production using microbial platforms (e.g., *E. coli*) are reducing costs, economic feasibility for a high-acreage crop like wheat remains a hurdle [39]. The species-specificity of our selected targets, particularly those with low homology to non-target organisms, is a significant advantage for regulatory compliance and reducing ecological impact [40]. A strategic benefit of targeting wing development is that it suppresses population spread and virus transmission without causing immediate mortality, potentially delaying the onset of resistance compared to lethal insecticides [41].

In conclusion, while practical hurdles exist, the pathway to commercialization is clearly delineated by ongoing innovations in RNAi technology. Our study provides the essential target gene validation required to initiate this development pipeline. The high specificity of the approach, combined with its potential for integrated pest management, positions it as a sustainable and promising future tool for controlling *R. padi*.

## Figures and Tables

**Table 1:** Specific primers for verifying some differentially expressed genes between winged and wingless *R. padi.*

Gene Name	Forward Primer (5′→3′)	Reverse Primer (5′→3′)
*BCORL1*	TCAAGGTAAGTTGTGCACCG	TGCATCCTCAGCAACACAGT
*AMP-L*	TTGACGAATCAATTAACCAGCATGA	AGCAATTTGATATTTGGAACAAACC
*Pfl*	GATCCGCTGCTTGGGAACTAT	GATCCGCTGCTTGGGAACTAT
*Lip3L*	GACGTTGTGGATCAACTGTGG	GCTATAACTCAACGCAGTGGC
*HLFL*(*X7*)	CTCTTGTCGAAGGCCCATGT	GAACAAGGCCGTTTTCCGAC
*HLFL*(*X4*)	AATCCTCAGGCAAAGCGTGT	GTTCTGATGGTCGCCTTCCA
*Actin*	TGGTATCGTCTTGGATTCTG	TTAGGTAGTCGGTGAGATCA

**Table 2:** GO enrichment of differentially expressed genes between winged and wingless morphs of *R. padi.*

GO.ID	Term	Ontology	Significant	Annotated	q Value
GO:0005214	structural constituent of chitin-based cuticle	molecular function	5/27	93/28478	5.78 × 10^−5^
GO:0042302	structural constituent of cuticle	molecular function	5/27	94/28478	5.78 × 10^−5^
GO:0008010	structural constituent of chitin-based larval cuticle	molecular function	4/27	70/28478	0.000799493
GO:0044421	extracellular region part	cellular component	10/36	1513/31556	0.0063394
GO:0005576	extracellular region	cellular component	12/36	2323/31556	0.0063394
GO:0040003	chitin-based cuticle development	biological process	6/34	292/32103	0.00968484
GO:0042335	cuticle development	biological process	7/34	532/32103	0.0100188

**Table 3:** KEGG enrichment analysis of differentially expressed genes between winged and wingless *R. padi.*

KO Number	Gene ID	Gene Symbol	Gene Function	TPM Value		q-Value	log_2_FC
				Winged Morph	Wingless Morph		
K03006	TRINITY_DN34565_c1_g2	*BCORL1*	Implication in parthenogenesis or embryonic patterning of aphids	148.83	564.05	<0.05	−1.92
K03006	TRINITY_DN30834_c1_g1	*AMP-L*	A potential role in aphid molting or reproduction processes	138.02	311.1	<0.05	−1.17
K14621	TRINITY_DN33233_c2_g1	*PLB1L*	Suggests the conservation of lipid exchange functions between aphids and their symbionts	23.51	56.39	<0.05	−1.26
K05759	TRINITY_DN34550_c1_g2	*Pfl*	Potentially involved in aphid parthenogenesis or embryonic development	27.89	57.52	<0.05	−1.04
K01052	TRINITY_DN27775_c0_g1	*Lip3L*	Associated with aphid nutritional adaptation strategies	1.63	0.18	<0.05	3.22
K09057	TRINITY_DN36053_c1_g1	*HLFL (X7)*	Regulates the ecdysteroid signaling pathway	19.54	64.14	<0.05	−1.71
K09057	TRINITY_DN36053_c1_g3	*HLFL (X4)*	Potentially associated with aphid developmental regulation	4.37	20.36	<0.05	−2.22

## Data Availability

The raw data supporting the conclusions of this article will be made available by the authors on request.

## Supplementary Materials

The following supporting information can be downloaded at: https://www.mdpi.com/article/10.3390/genes16101163/s1, Figure S1: Length distribution of Transcript and Unigene sequences; Table S1: Assembly statistics of transcriptomic data. Table S2: Statistics of gene annotation results.
