# Supplementary material for: Lipid Metabolism and Circadian Regulation in Wing Polyphenism of Rhopalosiphum padi: Transcriptomic Validation of Key DEGs for Biocontrol
Source: Genes (Basel). 2025 Sep 30;16(10):1163. doi: 10.3390/genes16101163 (PMC12564907; doi:10.3390/genes16101163)
Supplement: Supplementary file 1 [file genes-16-01163-s001.zip › genes-3897008-supplementary.pdf]

Table S1. Assembly statistics of transcriptomic data

	No.	>=500bp	>=1000bp	N50 (bp)	N90 (bp)	Max Len	Min Len	Total Len	Average Length
Transcript	231 824	132 972	87 320	1 902	431	12 570	201	251 363 981	1084.29
Unigene	108 632	37 967	18 108	1 077	263	12 570	201	72 408 051	666.54

Table S2. Statistics of gene annotation results

Database	Number of annotated genes	Percentage of annotated unigenes (%)
CDD	15382	14.16
PFAM	19035	17.52
KEGG	16988	15.64
KOG	23558	21.69
Swissprot	32490	29.91
GO	33491	30.83
NR	44093	40.59
NT	50955	46.91
Annotated in at least one database	72187	66.45
Annotated in all databases	2771	2.55
Total genes	108632	100

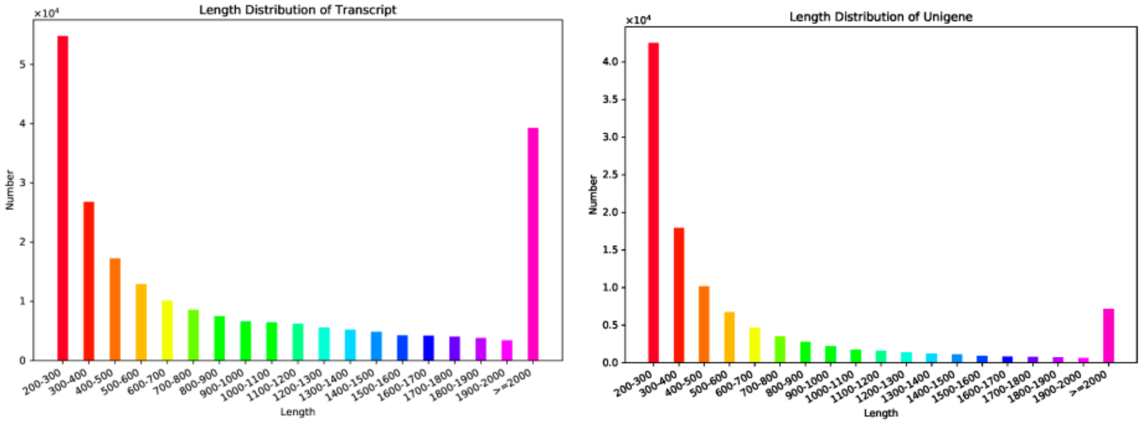

Figure S1. Length distribution of Transcript and Unigene sequences. The x-axis represents length intervals, and the y-axis indicates the number of sequences within each interval.
